# Prevalence and Predictors of Low Vitamin B6 Status in Healthy Young Adult Women in Metro Vancouver

**DOI:** 10.3390/nu8090538

**Published:** 2016-09-01

**Authors:** Chia-ling Ho, Teo A. W. Quay, Angela M. Devlin, Yvonne Lamers

**Affiliations:** 1 Food Nutrition and Health, Faculty of Land and Food Systems, The University of British Columbia, 2205 East Mall, Vancouver, BC V6T 1Z4, Canada; jennifer.ho@ubc.ca (C.-L.H.); quayt@mail.ubc.ca (T.A.W.Q.); 2 Research Institute, British Columbia Children’s Hospital, 950 West 28th Ave, Vancouver, BC V5Z 4H4, Canada; adevlin@cfri.ubc.ca; 3 Department of Pediatrics, The University of British Columbia, 4480 Oak Street, Vancouver, BC V6H 3V4, Canada; 4 Fraser Health Authority, 10334 152A St, Surrey, BC V3R 7P8, Canada

**Keywords:** vitamin B6, pyridoxal 5’-phosphate, suboptimal status, deficiency, ethnicity, dietary intake, supplement use, women, periconceptional

## Abstract

Low periconceptional vitamin B6 (B6) status has been associated with an increased risk of preterm birth and early pregnancy loss. Given many pregnancies are unplanned; it is important for women to maintain an adequate B6 status throughout reproductive years. There is limited data on B6 status in Canadian women. This study aimed to assess the prevalence of B6 deficiency and predictors of B6 status in young adult women in Metro Vancouver. We included a convenience sample of young adult non-pregnant women (19–35 years; *n* = 202). Vitamin B6 status was determined using fasting plasma concentrations of pyridoxal 5’-phosphate (PLP). Mean (95% confidence interval) plasma PLP concentration was 61.0 (55.2, 67.3) nmol/L. The prevalence of B6 deficiency (plasma PLP < 20 nmol/L) was 1.5% and that of suboptimal B6 status (plasma PLP = 20–30 nmol/L) was 10.9%. Body mass index, South Asian ethnicity, relative dietary B6 intake, and the use of supplemental B6 were significant predictors of plasma PLP. The combined 12.4% prevalence of B6 deficiency and suboptimal status was lower than data reported in US populations and might be due to the high socioeconomic status of our sample. More research is warranted to determine B6 status in the general Canadian population.

## 1. Introduction

Vitamin B6 (B6) has an obligatory role in the endocrine system, immune competence, and heme biosynthesis. In the form of pyridoxal 5’-phosphate (PLP), B6 serves as a coenzyme for >140 reactions in human metabolism, including in the interconversion of amino acids, synthesis of neurotransmitters, regulation of energy homeostasis, and formation of heme [1]. In one-carbon metabolism, PLP acts as a coenzyme for the glycine cleavage system, the interconversion of glycine and serine, and the transsulfuration pathway [2,3]. Other coenzyme functions of PLP include reactions in the kynurenine pathway that catabolizes tryptophan [4]. 

Maternal B6 adequacy is crucial at conception and throughout pregnancy to ensure healthy pregnancy outcomes. In a meta-analysis of maternal B6 interventions (*n* = 247), maternal B6 supplementation (2.6 to ≥50 mg/day) was associated with a 217 g higher infant birth weight [5]. A lower incidence of preeclampsia was reported in women supplementing with 10 mg/day of B6 during pregnancy compared to those who did not use supplements [6]. Low maternal B6 status has been associated with a lower Apgar score, an indicator of infant birth health assessed within minutes after delivery [7,8]. In a prospective cohort of 458 young Chinese women, women who experienced an early pregnancy loss had significantly lower periconceptional plasma PLP concentration compared to women with a healthy pregnancy outcome [9]. Also, low periconceptional B6 status (plasma PLP < 30 nmol/L) has been associated with a reduced probability of conception [10]. Given an estimated 50% of pregnancies are unplanned [11], it is important for women to maintain an adequate B6 status throughout reproductive years. 

Despite the crucial role of B6 in health, there is no nationally representative data on biochemical B6 status of Canadian women. The Canadian Community Health Survey (CCHS), Cycle 2.2, Nutrition Focus, in 2004 reported that 18% of Canadian adult women did not meet the Estimated Average Requirement (EAR; 1.1 mg/day) of B6 from dietary sources; however, there was no biochemical measurement of B6 status [12]. In the US National Health and Nutrition Examination Survey (NHANES), over 40% of young adult women (21–44 years) had B6 deficiency (defined as having plasma PLP concentration < 20 nmol/L; [13]). In light of these findings, there is an urgent need to investigate the B6 status of Canadian women. 

In several other countries (US, Ireland, Norway), various socioeconomic and lifestyle factors have been associated with plasma PLP concentrations, including oral contraceptive use [13], use of supplemental B6 [13,14], smoking [15,16], physical inactivity, and poverty [17]. Finding predictors of low B6 status in a Canadian population will contribute to the identification of vulnerable populations groups for low B6 status and related health consequences in Canada.

This study aimed to determine the prevalence of suboptimal B6 status and B6 deficiency in a convenience sample of young adult women in Metro Vancouver, using plasma PLP, a biochemical marker of B6 status. Demographic, dietary, and lifestyle predictors associated with plasma PLP were also assessed.

## 2. Experimental Section 

### 2.1. Participants

This study used data from a descriptive cross-sectional study conducted between 2012 and 2013. The original study was designed to determine the prevalence and predictors of low vitamin B12 status in a convenience sample of young adult women of South Asian and European descent. The recruitment and methods of the original study have been described in detail [18]. In brief, a total of 207 healthy, non-pregnant women aged 19 to 35 years of either South Asian or European descent living in Metro Vancouver were recruited by active and passive recruitment using paper and internet-based advertising, community outreach, and word of mouth. Eligibility of the potential research participants was determined by using a screening questionnaire that assessed multiple factors, including age, ethnicity, residence, and health condition to identify individuals who fit the target demographic. Informed written consent was obtained from all participants in the study. The UBC Clinical Research Ethics Board (H11-01216), Vancouver Coastal Health Research Ethics Board, and Fraser Health Research Ethics Board granted human ethics approval.

### 2.2. Blood Collection and Analysis

Fasting venous blood samples were obtained from all participants during a single clinic visit. Samples were collected into lithium heparin vacutainers. Plasma was separated using centrifugation and stored at –80 °C until analyses. Blood samples of 202 subjects were available for plasma PLP analysis. Plasma PLP was measured by quantification of its semicarbazide derivative using high-performance liquid chromatography (HPLC) with fluorescence detection [19]. The assay showed good agreement and high precision when measuring external controls at low, medium, and high PLP concentrations, with intra- and inter-assay coefficients of variation of 2.6%, 2.2%, and 2.3%, and 15.7%, 9.5%, and 10.2%, respectively. The cut-off values used for plasma PLP were: Plasma PLP > 30 nmol/L for adequate B6 status, plasma PLP = 20–30 nmol/L for suboptimal B6 status [20], plasma PLP < 20 nmol/L for B6 deficiency [20,21].

### 2.3. Demographic, Anthropometric, Exercise and Dietary Data

A demographic questionnaire, the “International Physical Activity Questionnaire (IPAQ)—Short Last 7 Day Self-Administered Format” [22,23], and a semi-quantitative Food Frequency Questionnaire (FFQ) “Your dietary intake” [24] were administered under the supervision of research staff during the clinic visit. 

The demographic questionnaire collected information on age, ethnicity, immigration, education, household income, oral contraceptive use, and supplement use. South Asian and European ethnicities were defined as having at least three grandparents from a single ethnic group. South Asian ethnicity included Bangladeshi, Bengali, East Indian, Goan, Gujarati, Hindu, Ismaili, Kashmiri, Nepali, Pakistani, Punjabi, Sikh, Sinhalese, Sri Lankan, and Tamil ethnic groups. Because of the low number of participants (*n* = 8) indicating secondary school education or less than secondary school education, education was dichotomized. Low education was considered as below a bachelor’s degree, and high education as equal or higher than bachelor’s degree. Due to the low number of participants (*n* = 36) in the lowest household income bracket, household income was dichotomized into the following categories: Low income, total annual household income < $30,000 if 1–2 people, <$40,000 if 3–4 people, <$60,000 if ≥5 people, and high income, total annual household income ≥ $30,000 if 1–2 people, ≥$40,000 if 3–4 people, ≥$60,000 if ≥5 people. Participants who reported use of oral contraceptives were classified as oral contraceptive (OC) users. Duration and frequency of OC use were recorded. Participants who reported current consumption of any nutritional vitamin or mineral supplements were classified as nutritional supplement users. Participants were asked to bring in nutritional supplement bottles. Brand names and frequencies of intake were recorded and B6 content (yes/no) was obtained retrospectively by researchers from web-based product information. Participants who were taking supplements containing B6 were classified as supplemental B6 users. Subjects were asked about their smoking habit and categorized as non-smoker, former smoker, current occasional smoker (1–9 cigarettes/day), current regular smoker (10–19 cigarettes/day) or current frequent smoker (≥20 cigarettes/day). Due to the high number of non-smokers (*n* = 174), smoking data were dichotomized to non-smoker and smoker (including current smokers and former smokers).

The IPAQ asked the participants to recall their physical activities in the past seven days categorized as: Walking, moderate-intensity activities, and vigorous intensity activities. Each activity was recorded with its frequency (days per week) and duration (time per day). Participants were categorized into three physical activity levels; low, medium, and high, according to the IPAQ analysis protocol [22].

A semi-quantitative FFQ was used to determine dietary B6 intake. The questionnaire contained 78 food items and has been validated to assess micronutrient intakes in the Canadian population [24,25]. 

Anthropometric measurements, including height, weight, and waist circumference, were taken by research staff during the clinic visit. Body mass index (BMI) was calculated based on weight and height. 

### 2.4. Statistical Analyses

The primary outcome of the presented analyses is B6 status and described using the direct biomarker plasma PLP and established cut-offs for adequate B6 status, suboptimal B6 status [20], and B6 deficiency [21]. Data were examined for normality by visual histogram assessment. Plasma PLP concentration was log-transformed to carry out statistical analyses and was presented as geometric mean (95% confidence interval (CI)). Relative dietary B6 intakes were presented as quartile ranges.

Bivariate analysis was conducted to identify variables associated with plasma PLP concentration (as continuous variable and as categorical variable using B6 status cutoffs) and relative dietary B6 intake. Two sample *t* tests were used for dichotomous variables, one-way ANOVA followed by Tukey’s Honest Significance test for categorical variables with more than two levels, and simple linear regression for continuous variables. If the *P* value from bivariate analysis was ≤0.2, the variable was carried forward to the stepwise multiple linear regression model. The full model with plasma PLP concentration as the dependent variable was controlled for relative dietary B6 intake, South Asian ethnicity, first generation immigrant status, BMI, education status, household income status, smoking status, and supplemental B6 use. Backward elimination procedure was used to establish the best fit multiple linear regression model. The estimated percentage change in plasma PLP concentration was presented for each variable after adjustment for other variables. Statistical significance was set at a two-sided *p* value of <0.05. All statistical analyses were performed using R software (3.1.2 windows version; R Foundation for Statistical Computing: Vienna, Austria).

## 3. Results 

### 3.1. Participant Characteristics

Overall, subjects (*n* = 202) were highly educated, with 71% having a bachelor’s degree or higher (Table 1). The rate of supplemental B6 use was 28% and did not differ based on age, BMI, ethnicity, household income, level of education, level of physical activity, OC use, or smoking status.

### 3.2. Blood Analyses

Mean (95% CI) concentration of plasma PLP was 61.0 (55.2, 67.3) nmol/L (Table 2). The prevalence of B6 deficiency (plasma PLP < 20 nmol/L) was 1.5% and that of suboptimal B6 status (plasma PLP = 20–30 nmol/L) was 10.9%. 

Users of supplemental B6 had significantly higher plasma PLP concentration compared to non-users of supplemental B6; mean (95% CI) plasma PLP concentrations were 48.5 (45.0, 52.3) and 111.5 (87.2, 139.9), respectively (*p* < 0.001). Women of South Asian descent had significantly lower plasma PLP concentration (47.6 (41.2, 54.8)) compared with women of European descent (66.9 (59.2, 75.6), *p* = 0.002). There was no significant difference in plasma PLP concentration based on education level, household income, physical activity level, OC use, or smoking status in bivariate analyses. There was no significant difference in the prevalence of B6 deficiency, suboptimal B6 status or both combined (plasma PLP < 30 nmol/L) compared to adequate B6 status based on any demographic or lifestyle factors.

### 3.3. Dietary Analyses

Quartiles of dietary B6 intake were 0–1.1 mg/day, 1.1–1.4 mg/day, 1.4–1.7 mg/day and >1.7 mg/day, respectively. Individuals with low household income had significantly lower dietary B6 intake compared to individuals with high household income (mean ± SD: 1.4 ± 0.42 vs. 1.5 ± 0.47, respectively; *p* = 0.045). Plasma PLP concentration and relative dietary B6 intake were positively but weakly correlated (*r* = 0.17; *p* = 0.02).

Dietary vitamin B6 intake derived mainly from meat and meat alternatives in this sample of young adult women of South Asian and European descent, as shown by multiple linear regression of dietary B6 intake (Table 3). There was no significant difference in the intake of meat and meat alternatives between women of South Asian and European descent (2.8 versus 3.0 servings; *p* = 0.2).

### 3.4. Multivariate Analyses

Relative dietary B6 intake, BMI, ethnicity and the use of supplemental B6 were significant predictors of plasma PLP concentration (Table 4). Relative dietary B6 intake and the use of supplemental B6 were positively associated with plasma PLP concentration; BMI and South Asian ethnicity were negatively associated with plasma PLP concentration. The model explained 32% (*R*²) of the variance in plasma PLP concentration.

Women taking supplemental B6 are expected to have ~113% higher plasma PLP after adjusting for relative dietary B6 intake, BMI and ethnicity (compared to ~127% higher plasma PLP in the unadjusted model) (Table 4). South Asian descent was associated with 21% lower plasma PLP concentration compared to women of European descent (*p* = 0.026), after adjusting for relative dietary B6 intake, BMI and the use of supplemental B6.

## 4. Discussion

Since the vitamin B6 status of Canadian women was previously unknown, and the relationship between demographic, dietary, and lifestyle factors and plasma PLP had not been assessed, we conducted a study of B6 status in a convenience sample of 202 healthy young Canadian adult women. We identified a combined prevalence of B6 deficiency and suboptimal B6 status of 12.4%. We also found that body mass index, South Asian ethnicity, relative dietary B6 intake, and the use of supplemental B6 were significant predictors of plasma PLP.

As defined by the Institute of Medicine, a plasma PLP concentration of <20 nmol/L corresponds with B6 deficiency [21]. The prevalence of B6 deficiency (1.5%) in this sample was much lower than the 40% prevalence of B6 deficiency reported for women aged 21–44 years in the NHANES 2003–2004 [13]. This large discrepancy may partly be due to the high socioeconomic status of the women in our study compared with participants of the NHANES, a representative population-based survey in the US. Low socioeconomic status has been associated with low B6 status [15,17] and overall poorer nutritional intake [26]. Education and income status accounted for 6% of the variation in plasma PLP in the NHANES [15]. The discrepancy between our study and the NHANES may also be attributed to different analytical methods employed for quantification of plasma PLP. The enzymatic assay used for the NHANES 2003–2004 samples has been reported to underestimate plasma PLP concentrations to a degree that would result in a doubling of the prevalence of B6 deficiency compared to the HPLC method we employed [27]. In the recent Alberta Pregnancy Outcomes and Nutrition (APrON) cohort study, none of the 119 pregnant women in the first trimester and only one out of 528 (0.2%) second-trimester pregnant women was B6 deficient; the prevalence of suboptimal B6 status was not reported [28]. Like our study, the APrON cohort is comprised of women of high socioeconomic status and the lack of B6 deficiency observed is likely due to the high rate (>94%) of multivitamin supplement use throughout pregnancy [28].

Suboptimal B6 status has been associated with an increased risk of several chronic diseases, including cardiovascular disease [29], colorectal cancer [30], breast cancer [31] and ovarian cancer [32]. In our study, 11% of our subjects had suboptimal B6 status. A cross-sectional study in Boston reported 18% of suboptimal B6 status among Puerto Rican women aged 45–75 years (*n* = 1236) [17]. The lower education level and income of subjects in that study may explain the higher prevalence of suboptimal B6 status compared with our study. In light of the limited data on B6 status in Canadians [12,28] and our finding of a substantial rate of suboptimal status in a cohort of healthy women of high socioeconomic status, a representative, population-based study to determine B6 status of Canadians is timely and crucial. We predict a higher prevalence of B6 deficiency and suboptimal B6 status would be observed in a representative sample of Canadian women compared to our study.

The use of supplemental B6 was a strong predictor of plasma PLP concentration both before and after multivariate adjustment. This is consistent with large scale studies that reported higher plasma PLP concentrations and a lower prevalence of B6 deficiency in nutritional supplement users [13] and supplemental B6 users [14] compared with non-users. In this sample, 48% of women used nutritional supplements (i.e., any type of vitamin or mineral supplement) of whom 58% were retrospectively categorized as supplemental B6 users. The prevalence of nutritional supplement use in this sample (48%) was higher than the prevalence reported in the CCHS in 2004 [33] with 35% of Canadian women aged 19–30 years reporting nutritional supplement use.

Relative dietary B6 intake was not a strong predictor of plasma PLP concentration both before and after multivariate adjustment. The weak association may partially be due to the use of a semi-quantitative FFQ. The validation study of the FFQ reported an 11% underestimation of relative dietary B6 intake compared to the use of dietary recalls [24]. Although the FFQ was validated to assess micronutrient intakes in the Canadian population [24], it is possible that it was unable to record certain ethnic specific foods. Further, under-reporting appears to be a factor, as a 1566 kcal/day average daily energy intake is lower than would be anticipated. In the NHANES 2003–2004, a stronger association between total B6 intake (from food sources and supplements combined) and plasma PLP (*r* = 0.32, *p* < 0.001) was observed [13]. We are unable to report quantitative intake of B6 from supplements due to limitations of secondary analysis. The weaker association between plasma PLP and dietary B6 intake in this study compared to the NHANES data might be explained by our data reflecting dietary B6 from food alone and not supplemental B6.

Our study is the first to report that women of South Asian descent may have lower plasma PLP concentrations compared with women of European descent. The difference in B6 status between South Asian and European women was not related to the consumption of different food sources of B6; meat and meat alternatives were the main dietary B6 sources in these healthy adult women. The negative association between South Asian ethnicity and plasma PLP concentrations remained significant after adjustment for relative dietary B6 intake, BMI, and supplemental B6 use. Although the South Asian population was only 56, compared to the European population of 146 in this study, the sample size was sufficient to give over 80% of power with a small effect size of 0.2 and significance level of 0.05 in the multiple linear regression model. Ethnicity has been shown to correlate with nutritional biomarker levels of other micronutrients [34].

Body mass index was inversely associated with plasma PLP concentration after adjustment for relative dietary B6 intake, supplemental B6 use, and South Asian ethnicity. An inverse relationship of BMI and plasma PLP was also reported in the NHANES; every 25% increase in BMI was associated with a 13% decrease in plasma PLP [15]. The volumetric dilution of the blood was suggested as a potential underlying mechanism for the linkage between increasing body mass and decreasing concentrations of B6 as well as other B-vitamins [35]. In addition, plasma PLP concentration has been inversely associated with circulating concentration of C-reactive protein (CRP), a marker of systemic inflammation that is elevated in obesity [36,37].

Compared to other studies, we did not find OC use to be a significant predictor of B6 status. In recent literature, lower-dose OC use was associated with low B6 status [38]. In the NHANES 2003–2004, plasma PLP concentration was also significantly lower in OC users compared to non-users [13]. It was suggested that this may be due to the disruption of tryptophan metabolism by estrogen, independent of B6 status [39]. In our study, the effect of OC use on plasma PLP concentration was small and we had insufficient sample size to detect a statistically significant difference. We also lacked power to detect associations with other variables including physical activity and smoking.

The predictors of B6 status we assessed explained only 32% of the variability in plasma PLP. Our results might be confounded by unexplained biological factors or genetic modifiers. One recent study reported some variants in tissue nonspecific alkaline phosphatase gene influenced plasma PLP concentration, but the clinical implications of these variants were unclear [40].

Some weaknesses of this study include the recruitment of a convenience sample of relatively healthy women of high education and the lack of data on genetic variants. Over 71% of our study participants obtained a bachelor’s degree or higher, where a comparatively lower proportion (35%) of women aged 25 to 64 in Metro Vancouver reported this high level of education, according to the National Household Survey [41]. We used plasma PLP as our single biomarker to assess B6 status since it is currently the only established biomarker with cut-offs to define B6 deficiency [21]. However, plasma PLP only reflects the circulating concentration of B6 and is influenced by several factors, such as inflammation, serum albumin and alkaline phosphatase concentrations [42]. The derivation of reference intervals for sensitive functional biomarkers, e.g., plasma cystathionine [43], is crucial for evaluation of intracellular B6 status. 

## 5. Conclusions

We report a 12.4% combined prevalence of B6 deficiency and suboptimal B6 status in healthy young adult women in Metro Vancouver (*n* = 202). Periconceptional B6 adequacy is crucial for healthy pregnancy outcomes; and women with higher BMI and South Asian ethnicity might be more likely to have low B6 status. The lower prevalence of B6 deficiency and suboptimal B6 status in these women compared to reports from representative samples in other countries may be due to the high socioeconomic status of these women. Given the central roles of B6 in key biological functions and health, more research is warranted to determine B6 status in the general Canadian population. 

## Figures and Tables

**Table 1:** Demographic and lifestyle characteristics of 202 healthy young adult women in Metro Vancouver.

Variables	Total (*n* = 202)
Age *, years	26.7 ± 4.2
Body mass index ^†^, kg/m²	22.7 (22.2, 23.1)
Waist circumference ^†^, cm	74.9 (73.7, 76.1)
Total energy intakes ^†,‡^, kcal/day	1566 (1504, 1629)
Relative dietary vitamin B6 intake ^‡^, mg/day	
Q1	0–1.1
Q2	1.1–1.4
Q3	1.4–1.7
Q4	>1.7
Ethnicity, *n* (%)	
European	147 (73)
South Asian	55 (27)
Education, *n* (%)	
Less than secondary school education	2 (1)
Secondary school diploma	5 (3)
Post-secondary education	51 (25)
Bachelor’s degree	87 (43)
University degree or >bachelor’s degree	57 (28)
Household income ^§^, *n* (%)	
Lowest	36 (20)
Lower-middle	44 (24)
Upper-middle	52 (28)
Highest	51 (28)
Physical activity level ^||^, *n* (%)	
Low	8 (4)
Medium	110 (55)
High	82 (41)
Use of oral contraceptives, *n* (%)	58 (29)
Use of nutritional supplements, *n* (%)	97 (48)
Use of supplemental vitamin B6, *n* (%)	56 (28)
Current or former smoker, *n* (%)	28 (14)

* Values presented as arithmetic mean ± SD. ^†^ Values presented as geometric mean (95% confidence interval). ^‡^ 18 samples were excluded due to plausible misreports in the food frequency questionnaire (*n* = 184). ^§^ Income quartiles are: Lowest: <$15,000 if 1–2 people, <$20,000 if 3–4 people, <$30,000 if ≥5 people, Lower-middle: $15,000–29,999 if 1–2 people, $20,000–39,999 if 3 or 4 people, $30,000 to 50,000 if ≥5 people, Upper-middle: $30,000–59,999 if 1–2 people, $40,000–79,999 if 3–4 people, $60,000–79,999 if ≥5 people, Highest: >$60,000 if 1–2 people, >$80,000 if ≥3 people. Only 183 subjects provided household income information. ^||^ Only 200 subjects completed physical activity questionnaire.

**Table 2:** Differences in plasma pyridoxal 5’-phosphate (PLP) concentration in 202 healthy young adult women by categories of demographic and lifestyle factors.

Variables	Plasma PLP concentration				
	Subjects *n*	Mean (95% CI) nmol/L	<20 nmol/L *n* (%) *	20–30 nmol/L *n* (%) *	>30 nmol/L *n* (%)
Overall	202	61.0 (55.2, 67.3)	3 (1.5)	22 (11)	177 (88)
Anemia					
Hb > 12 g/dL	166	62.0 (55.5, 69.2)	3 (1.8)	17 (10)	146 (88)
Hb < 12 g/dL	36	56.4 (45.0, 70.8)	0	5 (14)	31 (86)
Ethnicity					
European ^‡^	147	66.9 (59.2, 75.6) ^†^	2 (1.4)	14 (10)	131 (89)
South Asian	55	47.6 (41.2, 54.8)	1 (1.8)	8 (15)	46 (84)
Education					
Low	7	57.7 (28.5, 116.9)	0	2 (29)	5 (71)
High	195	61.1 (55.3, 67.5)	3 (1.5)	20 (10)	172 (88)
Household income					
Low	80	59.5 (52.1, 68.0)	1 (1.3)	7 (9)	72 (90)
High	103	63.6 (55.3, 73.0)	2 (1.9)	15 (13)	86 (83)
Physical activity level				
Low	8	44.4 (37.8, 52.3)	0	0	8 (100)
Medium	110	60.6 (53.0, 69.4)	2 (1.8)	13 (12)	95 (86)
High	82	63.8 (54.5, 74.8)	1 (1.2)	9 (11)	72 (88)
Oral contraceptive use				
Non-user	144	59.9 (53.5, 67.1)	2 (1.4)	17 (12)	125 (87)
User	58	63.6 (52.0, 77.8)	1 (1.7)	5 (9)	52 (90)
Supplemental vitamin B6 use				
Non-user ^‡^	146	48.5 (45.0, 52.3)	3 (2.1)	18 (12)	125 (86)
User	56	111.5 (87.2, 139.9) ^†^	0	4 (7)	52 (93)
Smoker				
No	174	61.1 (55.0, 67.8)	2 (1.2)	18 (10)	154 (89)
Yes	28	60.2 (44.0, 82.3)	1 (3.6)	4 (14)	23 (82)

Plasma PLP concentration was presented as geometric mean (95% confidence interval (CI)) and was log-transformed to carry out the following statistical analyses. Hb, hemoglobin. * Chi-squared test revealed no significant difference in the prevalence of B6 deficiency and suboptimal B6 status in all the variables. ^†^ *p* value < 0.05 for two-sample *t* test comparing the indicated category with the referent category. ^‡^ Referent category.

**Table 3:** Food sources of vitamin B6.

Variables	Adjusted change in dietary B6 intake, mg/day (95%CI)	Adjusted *p* value
Grains, serving	0.03 (0.01, 0.04)	<0.001
Fruits and Vegetables, serving	0.10 (0.08, 0.11)	<0.001
Dairy, serving	0.10 (0.07, 0.13)	<0.001
Meat and Alternatives, serving	0.19 (0.16, 0.22)	<0.001

Adjusted changes and *p* value were from multiple linear regression model controlled for intake of grains, fruits and vegetables, dairy, and meat and alternatives. Number of observation = 184, Model *p* value < 0.001, *R*²: 0.79, Adjusted *R*²: 0.79.

**Table 4:** Predictors of plasma PLP concentration from unadjusted and adjusted linear regression models.

Variables	Unadjusted % change in plasma PLP (95% CI) *	Unadjusted *p* value	Adjusted % change in plasma PLP (95% CI) *	Adjusted *p* value
Relative B6 intake				
Q2 (1.1–1.4 mg/day)	7.7 (–20.0, 45.0)	0.62	9.0 (–15.8, 41.2)	0.51
Q3 (1.4–1.7 mg/day)	27.5 (–5.3, 71.6)	0.11	23.1 (–4.4, 58.6)	0.11
Q4 (>1.7 mg/day)	35.6 (0.7, 82.4)	0.045	29.3 (0.3, 66.7)	0.048
BMI, kg/m²	–3.7 (–6.3, –1.0)	0.007	–2.7 (–5.1, –0.2)	0.034
South Asian descent	–28.9 (–33.6, –24.0)	0.002	–21.1 (–35.7, –3.1)	0.024
Supplemental B-6 use	127.7 (115.6, 140.8)	<0.001	114.5 (75.6, 162.0)	<0.001

Unadjusted percentage changes and *p* value were from simple linear regression models. Adjusted percentage changes and *p* value were from multiple linear regression model controlled for relative dietary B-6 intake, South Asian ethnicity, BMI, and supplemental B-6 use. Number of observation = 184, Model *p* value <0.001, *R*²: 0.32, Adjusted *R*²: 0.29. * The percentage change in plasma PLP concentration was calculated by ($e^{\beta 1}-1)$ × 100%.
